# CAREGIVERS' EXPECTATIONS OF A MIND-BODY THERAPY FOR COMPLICATED GRIEF

**DOI:** 10.1093/geroni/igac059.1129

**Published:** 2022-12-20

**Authors:** Harleah Buck

**Affiliations:** Csomay Center for Gerontological Excellence, Iowa City, Iowa, United States

## Abstract

Accelerated Resolution Therapy (ART) is a psychotherapy for the treatment of complicated grief, defined as unusually prolonged, functionally impairing grief. The purpose of this study was to qualitatively examine caregiver’s expectations of ART. The sample included 29 primarily female, older (67.4 + 7.1 years) former informal caregivers; a little over half (n=18) had been married to their care recipient. Thematic analysis resulted in three themes and six sub-themes arising: The role of knowledge in expectations (sub-themes uncertainty, prior knowledge); The role of personality in expectations (sub-themes openness, positive affect); and Expecting a process (sub-themes cognitive processes, affective processes) which described the interaction of person and process in shaping expectations of our intervention. An across theme analysis of the specificity of the participants’ expectations uncovered that knowledge and personality inform expectations of ART and that individuals who verbalize a process for recovery tend to be very specific in their expectations.

